# Giant Pancreatic Pseudocysts: Procedures in Management

**DOI:** 10.1155/1992/62109

**Published:** 1992

**Authors:** S. Paterson Brown, R. C. N. Williamson

**Affiliations:** Royal Postgraduate Medical School Department of Surgery Hammersmith Hospital, Du Cane Road London W12 0HS UK


*HPB Surgery*, 1992, Vol. 6, pp. 129–139
Reprints available directly from the publisher
Photocopying permitted by license only

© Harwood Academic Publishers GmbH
Printed in the United States of America

### HPB INTERNATIONAL

EDITORIAL & ABSTRACTING SERVICE

JOHN TERBLANCHE, EDITOR
Department of Surgery,
Medical School · Observatory 7925
Cape Town · South Africa
Telephone: (021) 47-1250 Ext. 2
Telefax (021) 448-6461

### GIANT PANCREATIC PSEUDOCYSTS: PROCEDURES IN MANAGEMENT

### ABSTRACT

*Johnson, L.B., Rattner, D.W. and Warshaw, A.L. The effect of size of giant pancreatic pseudocysts on the outcome of internal drainage procedures. Surgery Gynecology & Obstetrics* 173, *111—174.*

**Pancreatic pseudocysts (PP) that fail to resolve spontaneously are optimally treated by internal drainage to a viscus. Pseudocysts adherent to the stomach are usually drained by way of cystgastrostomy. Recent experience with giant pseudocysts (> 15 centimeters), however, challenges this approach. Fifty-two patients with pancreatic pseudocysts of various sizes were treated from 1982 to 1986 at the Massachusetts General Hospital. Twenty-eight PP were suitable for internal drainage. The postoperative complication rate was directly proportional to the size of the pseudocyst. Four patients had giant PP, three of which occurred after an attack of acute pancreatitis. All four were treated by cystgastrostomy.**

**Three of four patients with giant pseudocysts had life-threatening postoperative complications as a result of incomplete emptying of the cyst, and two patients died. No evidence of anastomotic leakage could be demonstrated by upper gastrointestinal series or computed tomographic scans. Transgastric drainage tubes in these three instances were not protective. We conclude that cystgastrostomy may not be appropriate for the treatment of giant pancreatic pseudocysts because it fails to provide dependent drainage of a large cyst cavity.**

**If internal drainage is performed, the cyst should be anastomosed to a defunctionalized loop of jejunum in a dependent position. In some instances, external drainage of giant pancreatic pseudocysts may be safer than cystgastrostomy.**

### PAPER DISCUSSION

KEY WORDS: Pancreatitis, pancreatic pseudocyst

The data from this paper by Dr Warshaw and colleagues at the Massachusetts General Hospital are open to a number of interpretations. Among 52 patients with pancreatic pseudocyst seen over a five-year period, four with giant cysts (> 15 cm diameter) did badly: three developed serious postoperative complications and two died. Since all four patients had received a cystgastrostomy, the authors draw the reasonable conclusion that this operation is inappropriate for large pancreatic pseudocysts, although the numbers are too small for a clearcut verdict. A stricter interpretation of the data from this study and its predecessors suggests that there are several contraindications to cystgastrostomy, as follows:

1. Small pseudocysts complicating acute pancreatitis. Although only nine cysts in the present series resolved spontaneously, it can surely be assumed that most of these belonged to the acute group ($n = 18$). Pseudocysts that arise in chronic pancreatitis are much less likely to undergo spontaneous resolution unless they follow an acute flare-up of the disease and the pancreatic duct is not involved[1,2]. A small transient pseudocyst is quite a common complication of acute pancreatitis and does not need formal internal drainage. Another study has indicated that up to 50 per cent of all pseudocysts will resolve, including 27 per cent of those > 10cm diameter[1]. Thus an observational policy is appropriate, at least for the first few weeks. Although seven patients with acute pseudocysts in the present series were treated by external operative drainage, percutaneous drainage seems a better option for those who develop symptoms during the waiting period. Good results can be obtained in 90 per cent of cases, even if the cyst communicates with the pancreatic duct[2], though the presence of concomitant pancreatic necrosis greatly reduces the chance of success[3].

2. Most pseudocysts complicating chronic pancreatitis. In our experience these cysts tend to be contained within the capsule of the pancreas, and the lesser sac is often patent, i.e. the cyst is not adherent to the back of the stomach. Cystgastrostomy is therefore inappropriate in such cases, whether it is performed endoscopically or by open operation. Smaller cysts in the body and tail of pancreas may best be treated by distal resection, as performed successfully in 11 patients in the present series. A small cyst in the head may be amenable to endoscopic cystduodenostomy. Larger cysts can be managed by cystjejunostomy, but it is important to delineate the pancreatic duct,[2] which may also require drainage into the Roux loop (unless it communicates with the cyst).

3. Larger cysts complicating acute pancreatitis, for the reasons outlined by Drs Johnson, Rattner and Warshaw.

There are theoretical reasons for preferring cystjejunostomy to cystgastrostomy when an internal drainage procedure is indicated. First, a Roux loop can be used to provide dependent drainage wherever the pseudocyst is situated. Second, the consequences of anastomotic dehiscence are likely to be less severe (there was one such leak following cystjejunostomy in the present series and it healed with conservative management). Third, the entry of gastric contents into an acutely inflamed pseudocyst cavity might provoke haemorrhage. However, cystgastrostomy is simpler, quicker and involves only one anastomosis. Few series have compared the two procedures. In one recent study, cystjejunostomy took longer to perform than cystgastrostomy but had a slightly lower rate of postoperative complications including bleeding[4]; the cyst recurrence rate was virtually identical (8 per cent versus 10 per cent). The same tendency towards a higher complication rate after cystgastrostomy emerges from three previous reports[6–8].

In summary, cystgastrostomy is an appropriate procedure for 'mature' acute pseudocysts that are large enough to cause symptoms but not so large as to prevent adequate drainage by this approach. For the rest (probably the majority), cystjejunostomy offers a safer alternative at the expense of a longer operation.

### REFERENCES

1. Yeo, C.J., Bastidas, J.A., Fishman, E.K., Zinner, M.J. and Cameron, J.L. (1990) The natural history of pancreatic pseudocysts documented by computed tomography. *Surg. Gynecol. Obstet.*, **170**, 411–417
2. D'Egidio, A. and Schein, M. (1991) Pancreatic pseudocysts: a proposed classification and its management implications. *Br. J. Surg.*, **78**, 981–984
3. Vansonnenberg, E., Wittich, G.R. and Casola, G. *et al.* (1989) Percutaneous drainage of infected and noninfected pancreatic pseudocysts. *Radiology*, **170**, 757–762
4. Steiner, E., Mueller, P.R. and Hahn, P.F. *et al.* (1988) Complicated pancreatic abscesses: problems in interventional management. *Radiology*, **167**, 443–446
5. Newell, K.A., Liu, T., Aranha, G.V. and Prinz, R.A. (1990) Are cystgastrostomy and cystjejunostomy equivalent operations for pancreatic pseudocysts? *Surgery*, **108**, 635–639
6. Frey, C.F. (1978) Pancreatic pseudocysts — operative strategy. *Ann. Surg.*, **188**, 652–662
7. Schattenkerk, M.E., Vries, J.E., Bruining, H.A., Eggink, W.F. and Obertop, H. (1982) Surgical treatment of pancreatic pseudocysts. *Br. J. Surg.*, **69**, 593–594
8. Kohler, H., Schafmayer, A., Ludtke, F.E., Lepsien, G. and Peiper, H.J. (1987) Surgical treatment of pancreatic pseudocysts. *Br. J. Surg.*, **74**, 813–815

S. Paterson Brown
and R. C. N. Williamson
Royal Postgraduate Medical School
Department of Surgery
Hammersmith Hospital
Du Cane Road
London W12 0HS, UK

### EFFECT OF INTRAOPERATIVE HYPOTENSION ON SURVIVAL AFTER RESECTION OF COLORECTAL LIVER METASTASES

### ABSTRACT

*Younes, R.N., Rogatko, A. and Brennan, M.F. (1991) The influence of intraoperative hypotension and perioperative blood transfusion on disease-free survival in patients with complete resection of colorectal liver metastases. Annals of Surgery,* **214**, *107-113.*

